# Multi-level gene expression profiles affected by thymidylate synthase and 5-fluorouracil in colon cancer

**DOI:** 10.1186/1471-2164-7-68

**Published:** 2006-04-03

**Authors:** Yaguang Xi, Go Nakajima, John C Schmitz, Edward Chu, Jingfang Ju

**Affiliations:** 1 Mitchell Cancer Institute, University of South Alabama, Mobile, AL, 36688, USA; 2 Yale Cancer Center, Yale University School of Medicine, New Haven, CT 06501, USA

## Abstract

**Background:**

Thymidylate synthase (TS) is a critical target for cancer chemotherapy and is one of the most extensively studied biomarkers for fluoropyrimidine-based chemotherapy. In addition to its critical role in enzyme catalysis, TS functions as an RNA binding protein to regulate the expression of its own mRNA translation and other cellular mRNAs, such as p53, at the translational level. In this study, a comprehensive gene expression analysis at the levels of both transcriptional and post-transcriptional regulation was conducted to identify response markers using human genome array with TS-depleted human colon cancer HCT-C18 (TS-) cells and HCT-C18 (TS+) cells stably transfected with the human TS cDNA expression plasmid.

**Results:**

A total of 38 genes were found to be significantly affected by TS based on the expression profiles of steady state mRNA transcripts. However, based on the expression profiles of polysome associated mRNA transcripts, over 149 genes were affected by TS overexpression. This indicates that additional post-transcriptionally controlled genes can be captured with profiling polysome associated mRNA population. This unique approach provides a comprehensive overview of genes affected by TS. Additional novel post-transcriptionally regulated genes affected by 5-fluorouracil (5-FU) treatment were also discovered via similar approach.

**Conclusion:**

To our knowledge, this is the first time that a comprehensive gene expression profile regulated by TS and 5-FU was analyzed at the multiple steps of gene regulation. This study will provide candidate markers that can be potentially used for predicting therapeutic outcomes for fluoropyrimidine-based cancer chemotherapy.

## Background

Thymidylate synthase (TS) is a folate-dependent enzyme that catalyzes the reductive methylation of dUMP by 5,10-methylenetetrahydrofolate to form dTMP and dihydrofolate [1,2]. Because the TS-catalyzed enzymatic reaction provides the sole intracellular *de novo *source of thymidylate, an essential precursor for DNA biosynthesis, this enzyme has been an important target for cancer chemotherapy for the past 50 years [3-5]. TS is also one of the most extensively investigated biomarkers in recent years [6-10]. In certain cases, TS has been shown to be a significant biomarker for predicting patient responses to 5-FU based therapy. However, in other studies, the expression level of TS alone is not sufficient for clinical prognosis. The goal of this study is to provide global comprehensive gene profiles and networks at multiple levels of gene regulation that are affected by endogenous levels of TS protein and 5-FU administration. This information will provide the basis to identity biomarker gene candidates that can be further validated using clinical samples for future clinical diagnosis and prognosis.

The rational for investigating genes affected by TS and 5-FU at both transcriptional and post-transcriptional levels is that TS, in addition to its critical enzymatic function, functions as a RNA binding protein [11]. The translation of human TS mRNA is regulated by its own protein product via a negative autoregulatory mechanism whereby the binding of TS protein to at least two distinct sequences on its own TS mRNA results in translational repression [12,13]. TS is also capable of interacting with several other cellular mRNAs such as p53 mRNA and c-Myc mRNA [14,15]. Previous studies demonstrated that TS protein regulates p53 gene expression at least in part, at the translational level [16]. In this case, TS may be involved in coordinating the regulation of expression and/or function of cellular growth and proliferation and it is conceivable that TS may play an essential role as a regulator of cell cycle related events. More importantly, this study will have direct clinical relevance in that the mechanism of acute and long term 5-FU related drug resistance is distinct. The acute induction of TS expression after 5-FU treatment was regulated at the translational level and long term resistance for 5-FU is related to transcriptional activation and gene amplification of TS [17]. Thus, it would be particularly important to systematically investigate other potential post-transcriptional regulated genes via TS protein. This may be especially vital for the discovery of additional chemotherapeutic response related markers that otherwise would be missed by simply profiling steady state total mRNAs [18]. A recent report suggested that TS may also function as an oncogene to transform NIH3T3 cells [19]. A comprehensive gene expression profiling analysis may also gain new insights into signaling pathways that were deregulated by over-expression of TS. In this regard, it is critical to develop more comprehensive molecular expression profiles to provide candidate genes that can potentially be used for predicting clinical outcomes for colorectal cancer.

In this study, a systems biology approach was used to investigate genes altered by the overexpression of TS at both transcriptional and post-transcriptional levels using human genome expression array in TS-depleted human colon cancer HCT-C18(TS-) cells and HCT-C18 (TS+) cells stably transfected with the human TS cDNA expression plasmid. In addition, both cell lines were treated with 5-FU for different time periods in an attempt to identify both acute response genes as well as delayed response genes affected by the 5-FU exposure. A number of genes were identified by comparing the expression profiles obtained from both steady state total mRNAs and polysome associated mRNA transcripts isolated from 5-FU treated HCT-C18 (TS-) and HCT-C18 (TS+) cells. Comprehensive gene lists were generated that were relevant for both TS dependent and independent cytotoxicity by 5-FU exposure. We also discovered genes and pathways that may be involved for the oncogenic function of TS.

## Results

### Characterization of HCT-C18 (TS-) and HCT-C18 (TS+) cells

The HCT-C18 (TS-) cell line contains a missense mutation at amino acid 216 of the TS protein that results in a near complete inactivation of TS enzyme activity [20]. HCT-C18 (TS+) cells were stably transfected with an expression construct containing full length human TS cDNA. HCT-C18 (TS+) cells were able to grow without thymidine supplement in the media [16,20]. However, HCT-C18 (TS-) cells require 10 μM thymidine in RPMI1640 medium. The doubling times of both cells were identical at 24 hrs.

Previous study has demonstrated that the mechanism for the decreased p53 expression occurred at the translational level [16]. We use p53 as a positive control gene for monitoring our high throughput expression profiling approach. Our microarray gene expression analysis indicates that there is no difference in the expression level of p53 based on steady state total mRNA (Additional file 1). However, the expression level of actively translated p53 mRNA in HCT-C18 (TS+) cells was found to decrease by 4.5-fold compared to the p53 mRNA level in HCT-C18 (TS-) cells (Additional file 2). This is consistent with our previous results that p53 was regulated at the translational level via TS [16].

### Effect of 5-FU on the expression of TS and p53

**Figure 1:**
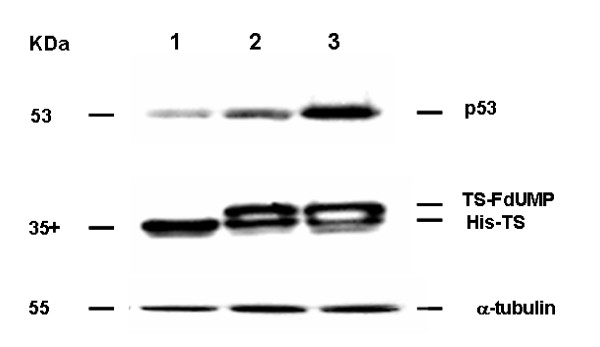
Effect of 5-FU on p53 and TS expression via Western immunoblot analysis. Equal amounts (25 μg) of protein extracts from both control and 5-FU treated HCT-C18 (TS+) cells were loaded and resolved with 10%

The effect of 5-FU on the expression of both TS and p53 was investigated using Western immunoblot analysis. HCT-C18 (TS+) cells were treated with 10 μM 5-FU for 4 hrs and 24 hrs. TS protein expression was increased by nearly 4-fold (lane 2) at 4 hrs and 10-fold at 24 hrs (lane 3) (Figure 1). The TS-FdUMP-tetrahydrofolate covalent ternary complex was clearly visible after 5-FU treatment. The expression of wild type p53 was also increased in response to 5-FU treatment (Figure 1, lane 2–3).

### Effect of TS on steady state total mRNAs expression

**Figure 2:**
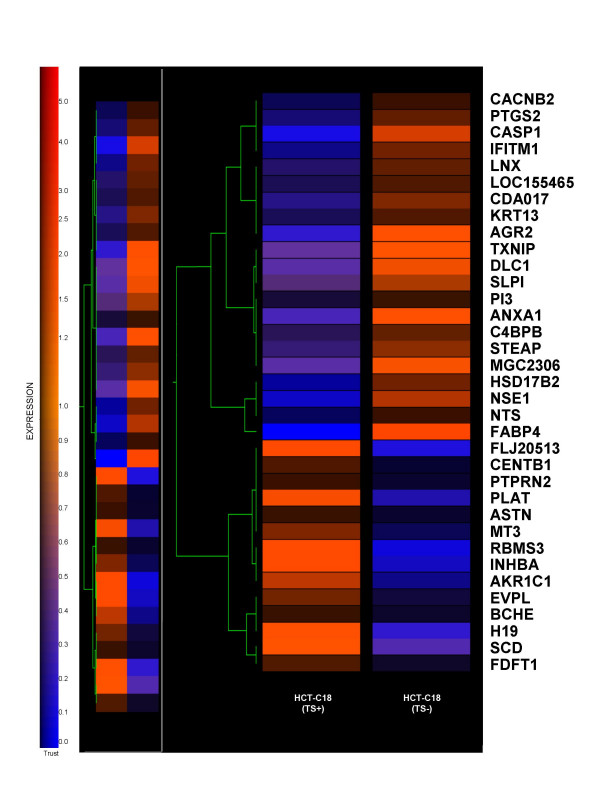
Clustering analysis of genes expression affected by TS overexpression with HCT-C18 (TS+) (Lane 1) vs. HCT-C18 (TS-) (Lane 2) based on the profiles of steady state total mRNAs. One-way ANOVA analysis was used for the gene clustering with p < 0.05.

The global gene expression profile of HCT-C18 (TS-) and HCT-C18 (TS+) cells based on steady state total mRNA expression was analyzed using human high density CodeLink oligo array (20 K). Although the growth characteristics of the two cell lines are similar in terms of doubling time, many genes are already affected by overexpressing TS protein. Our analysis revealed that over 38 genes were changed in their expression in response to TS expression (n = 3, p < 0.05 and 4-fold cut-off). The partial gene list and the clustering analysis are shown in Figure 2 (For complete gene list see Additional file 2). Genes such as CENTB1 and MT3, which are involved in cell proliferation and signaling, were up-regulated by TS. CASP1, a gene involved in regulation of I-κB/NF-κB cascade, apoptosis and signal transduction, was decreased 22-fold by TS overexpression.

### Effect of TS on polysome associated mRNAs

**Figure 3:**
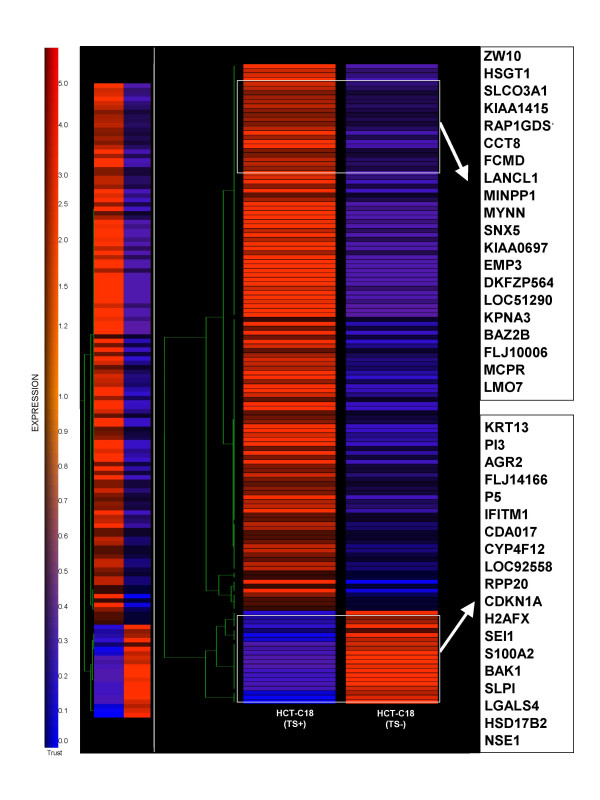
Clustering analysis of genes expression affected by TS overexpression with HCT-C18 (TS+) (Lane 1) vs. HCT-C18 (TS-) (Lane 2) based on the profiles of polysome associated mRNAs. One-way ANOVA analysis was used for the gene clustering with p < 0.05.

To discover novel genes post-transcriptionally regulated by TS protein, we isolated the polysome associated mRNAs from both HCT-C18 (TS-) and HCT-C18 (TS+) cells and performed expression analysis using human high density CodeLink oligo array (20 K). Over 149 genes were effected in TS overexpressing HCT-C18 (TS+) cells (n = 3, p < 0.05 with 4-fold cut-off) (Figure 3; Additional file 2). Genes involved in protein biosynthesis (FLJ10989, PEX1, FLJ20450, PSMC6, MRPL3) and cell cycle control (MAD2L1, INHBA, APPL, D123) were up-regulated at the post-transcriptional level. The overlapping genes (such as INHBA, and CASP1) between steady state total mRNA and polysome associated mRNA profiles are listed in Table 1.

### Steady State mRNAs affected by 5-FU treatment in HCT-C18 (TS+) Cells

**Figure 4:**
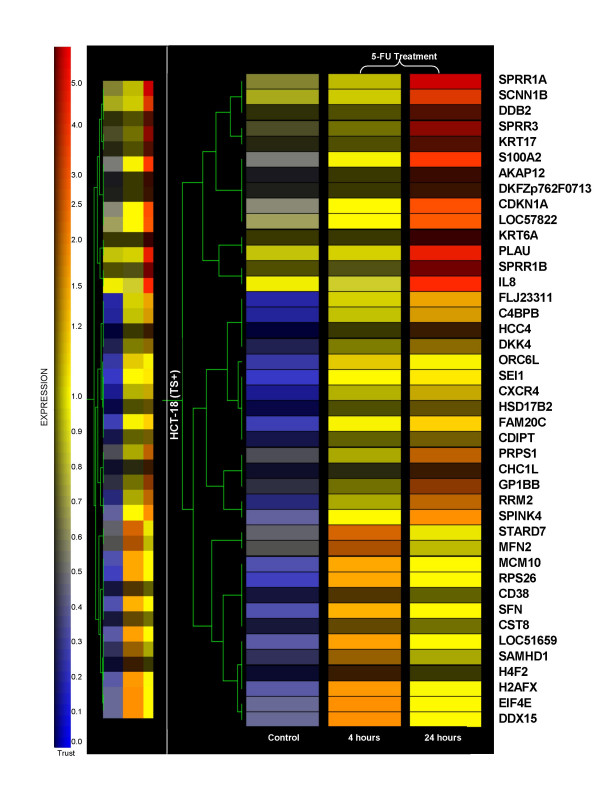
Clustering analysis of TS dependent cytotoxicity in HCT-C18 (TS+) cells (Lane 1) treated with 10 μM 5-FU for 4 hrs (Lane 2) and 24 hrs (Lane 3) based on the expression profiles generated from steady state total mRNAs via One-way ANOVA analysis with p < 0.05.

To identify acute and delayed response genes following 5-FU treatment in HCT-C18 (TS+) cells, gene expression profiling analysis was performed on steady state total mRNAs isolated from control and 5-FU treated samples at 4 hrs and 24 hrs time points. The expression analysis reveals that over 46 genes were affected by 5-FU treatment by One-way ANOVA analysis (n = 3, p < 0.05 with 4-fold cut-off). The clustering analysis is shown in Figure 4. (For complete gene list see Additional file 3). The expression analysis clearly showed a different gene expression profiles between 4 hrs and 24 hrs 5-FU exposure. Based on the comparison of the expression profiles among the HCT-C18 (TS+) control and 5-FU treated samples, a number of genes were acutely increased in response to 5-FU. Genes (shown in orange color in Figure 4, lane 2) such as ORCL6 (DNA replication), PRPS1 (nucleotide biosynthesis), DDX15 (mRNA splicing), DKK4 (Wnt signalling pathway), and eIF4E (regulation of protein biosynthesis) are some of the important genes affected by the acute 5-FU treatment based on our model. Expression of some of the genes, such as SPRR1A, DDB2, and CDKN1A, was only increased in response to 5-FU at 24 hrs (Figure 4. orange color in lane 3).

### Polysome associated mRNA Profiles with 5-FU treatment in HCT-C18 (TS+) cells

**Figure 5:**
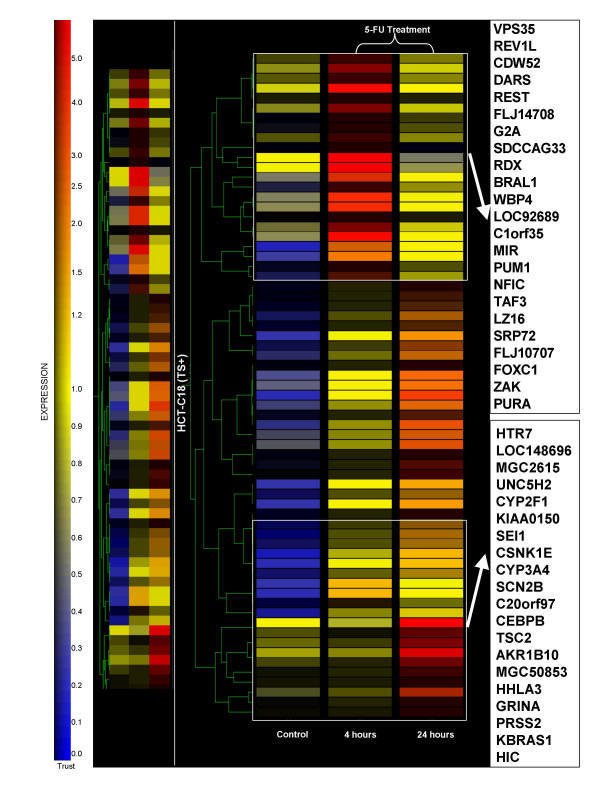
Clustering analysis of TS dependent cytotoxicity in HCT-C18 (TS+) cells (Lane 1) treated with 10 μM 5-FU for 4 hrs (Lane 2) and 24 hrs (Lane 3) based on the expression profiles generated from polysome associated mRNAs via One-way ANOVA analysis with p < 0.05.

To identify acute and delayed response genes that were affected at post-transcriptional level by 5-FU treatment, polysome associated mRNAs were isolated from control, and 5-FU treated samples at 4 hrs and 24 hrs. Gene expression analysis via microarray revealed that over 67 genes were affected in response to 5-FU (n = 3, p < 0.05 with 4-fold cut off). The clustering analyses are shown in Figure 5 (For complete gene list see Additional file 4). Gene expression analysis revealed dynamic changes in expression at 4 hrs and 24 hrs of 5-FU treatment. By comparing the expression profiles between control HCT-C18 (TS+) and 5-FU treated samples, we discovered additional post-transcriptionally controlled genes that otherwise would be missed if the expression analysis was only performed using steady state total mRNAs. Genes (Red color in Figure 5, lane 2, and lane 3) such as NFIC (DNA replication), SRP72 (protein phosphorylation; signal transduction), TSC2 (cell growth), IREB2 (translational regulator), and CEBPB (acute-phase response) are some of the genes that were regulated at the post-transcriptional level in response to 5-FU exposure with unique expression dynamics.

### Genes associated with 5-FU induced TS independent cytotoxicity

**Figure 6:**
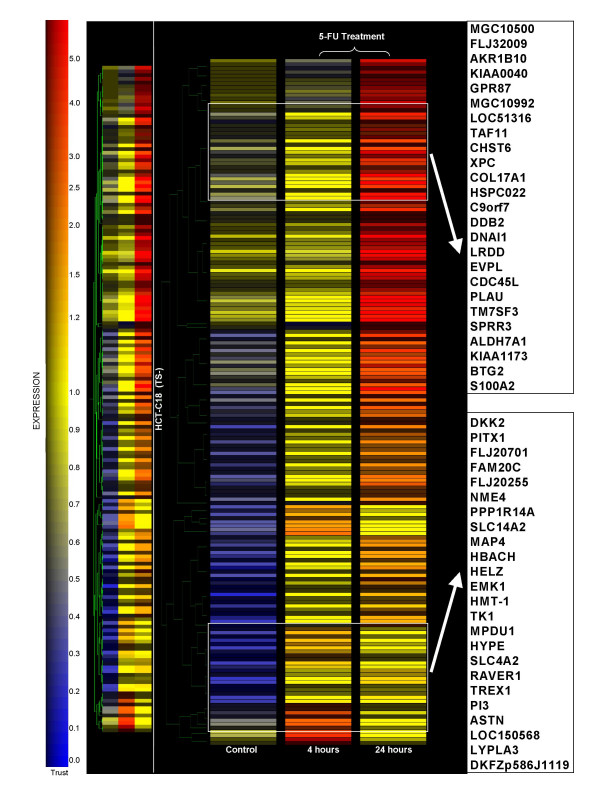
Clustering analysis of predictive marker genes for TS independent cytotoxicity in HCT-C18 (TS-) cells (Lane 1) treated with 10 μM 5-FU for 4 hrs (Lane 2) and 24 hrs (Lane 3) via One-way ANOVA analysis with p < 0.05.

To identify genes associated with TS independent cytotoxicity affected by 5-FU treatment, HCT-C18 (TS-) cells were treated with 10 μM 5-FU for 4 hrs and 24 hrs in the presence of 10 μM thymidine. The presence of thymidine provides the essential precursor for DNA biosynthesis for the HCT-C18 (TS-) cells thereby preventing the TS mediated cytotoxicity caused by 5-FU. In this case, the cytotoxicity of 5-FU in HCT-C18 (TS-) cells was a result of direct incorporation of 5-FU metabolites to DNA and RNA. Based on the clustering analysis of steady state total mRNA profiling of control HCT-C18 (TS-) cells and 5-FU treated samples in the presence of 10 μM thymidine, the gene expression profiles generated from these samples are mainly associated with TS independent toxicity in response to 5-FU by direct incorporation to RNA and DNA. 185 genes (n = 3, p < 0.05 with 4-fold cut-off) were affected. The clustering analysis is shown in Figure 6 (For complete gene list see Additional file 5).

**Figure 7:**
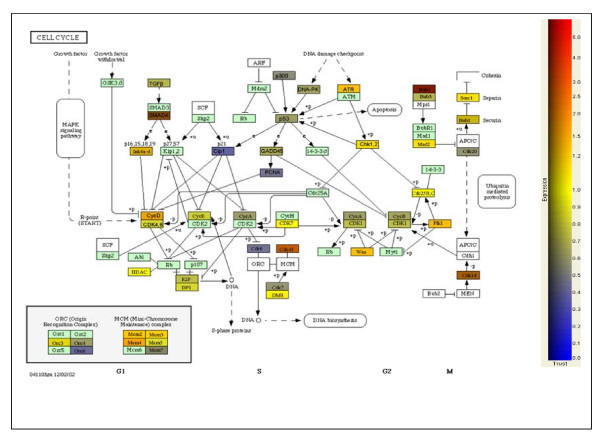
Real time pathway analysis of cell-cycle related genes mediated via TS overexpression in HCT-C18 (TS+) cells using actual gene expression data. (Red color indicates overexpression. Blue color indicates decreased expression).

To further define the genes that were associated with TS independent toxicity, we compared the expression clusters (Figures 4 and 6) from both HCT-C18 (TS-) and HCT-C18 (TS+) cells treated with 5-FU using Venn diagram analysis. The overlapping gene list is shown in Table 2. Genes such as RRM2 (DNA replication), CDKN1A (G1 arrest), SEI1 (cell proliferation), DDB2 (nucleotide-excision repair), SFN (cell cycle regulation) were associated with TS independent cytotoxicity to 5-FU treatment. Gene expression analysis from Figure 4 was used for the construction of the cell cycle control pathway after 5-FU treatment illustrated in Figure 7.

## Discussion

In this study, a systems biology approach was used to analyze global gene expression and its networks affected by TS and 5-FU at multiple levels of control. To the best of our knowledge, this is the first report to address genes that are affected at both transcriptional and post-transcriptional levels affected by elevated TS protein expression and 5-FU treatment. We believe that this will provide a better understanding of complex network related to the multi-functions of TS protein as an RNA binding protein or as a potential oncogene. It also provides a technology platform to systematically discover potential post-transcriptionally controlled genes by TS and 5-FU treatment. As our comparative model, we used a pair of human colon cancer cell lines: the mutant HCT-C18(TS-) subline in which the TS protein had been rendered marginally active and the TS over-expressing subline, HCT-C18(TS+), created by stable expression of a human TS cDNA plasmid. Both cell lines express wild-type p53 protein [16]. p53 is one of the known genes affected by the over-expression of TS at the translational level due to its RNA binding function [16]. Using our expression profiling approach, the levels of p53 mRNA are not significantly changed based on steady state total mRNA profile. However, the polysome associated p53 mRNA was decreased by 4.5-fold in HCT-C18 (TS+) cells compared with HCT-C18 (TS-) cells (listed in Additional file 2). These results, taken together, confirmed that our comprehensive gene profiling analysis approach is capable of discovering new post-transcriptionally regulated genes.

The gene profiling analysis based on steady state total mRNA transcripts revealed over 38 genes that were deregulated by TS over-expression (Figure 2 and Additional file 1). Although this study does not provide any direct evidence for TS as a cell cycle regulator, a number of cell cycle control and apoptotic control genes were perturbed by TS over-expression. One of the genes is inhibin/activin A (INHBA) that was up-regulated by nearly 22-fold in HCT-C18 (TS+) cells. INHBA is a growth factor that is involved in cell proliferation [21]. INHBA does this through type I and type II receptor serine kinases [21]. This may be a potential pathway by which TS is able to influence the cellular transformation process [21-23]. In fact, it has been reported recently that INHBA is over-expressed in stage IV colorectal cancer [24]. Additionally, single stranded RNA interacting protein (RBMS3), a gene responsible for mRNA splicing, was also up-regulated by 28-fold. The gene is a member of a small family of proteins which bind single stranded DNA/RNA. These proteins are characterized by the presence of two sets of ribonucleoprotein consensus sequence (RNP-CS) that contain conserved motifs, RNP1 and RNP2, originally described in RNA binding proteins, and required for DNA binding. These proteins have been implicated in such diverse functions as DNA replication, gene transcription, cell cycle progression and apoptosis [25]. The encoded protein was isolated by virtue of its binding to an upstream element of the alpha2 (I) collagen promoter. The observation that this protein localizes mostly in the cytoplasm suggests that it may be involved in a cytoplasmic function such as controlling RNA metabolism, rather than transcription. Thus, RBMS3 may play an important role as part of the post-transcriptional control mediated by TS. Conversely, genes such as caspase 1 (CASP1) that control cell cycle arrest and apoptosis, were down-regulated some 22-fold. Hence, future studies will focus on determining whether TS protein regulates the expression of these genes directly by binding to the mRNAs or indirectly through downstream mechanisms.

Using our systems biology approach of polysome associated mRNA comparisons, we have identified a list of genes that are over- or under-expressed due to TS protein over-expression (Figure 3 and gene list in Additional file 2). Many of them play key roles in cell-cell signalling, protein biosynthesis, RNA processing, DNA repair, cell cycle control and translational regulation. Some of them are previously known translational controlled genes such as EGFR [26], one of the key targets for the latest targeted anticancer drug development for colon cancer treatment [27,28]. In this study, the expression of EGFR was found to increase by nearly 8-fold by TS over-expression. Many other genes on this list are novel potential post-transcriptionally regulated genes. Among the down regulated genes, cyclin dependent kinase inhibitor p21 (CDKN1A) was decreased by over 5-fold. Apoptosis inhibitor BAK1 was also decreased by 6-fold in TS over-expressing cells. It seems that over expressing TS has set the stage for cells to have many growth advantages using multiple mechanisms.

To identify genes regulated at both transcriptional and post-transcriptional level, we performed an overlapping analysis by comparing the gene lists generated from steady state total mRNAs and polysome associated mRNAs using Venn diagram. There are 15 overlapping genes between the two lists (Table 1). D123 (CdC123), up-regulated by 5-fold in HCT-C18 (TS+) cells, has been shown to be regulated at the translational level via eIF2γ [29]. We have identified an additional 136 genes based on polysome associated mRNAs which could be easily missed if only steady state mRNA profiling is performed. This approach provides a technical platform to systematically analyzing gene expression at post-transcriptional level. It also reveals the importance of analyze gene expression at multiple levels.

Based on the clinical observation and detailed molecular investigations [11,17], TS can be acutely induced with a short time 5-FU exposure and the mechanism of TS induction was regulated, at least in part, at the translational level [11]. With this notion, we designed a strategy that allows us to capture both acute and delayed response genes in response to 5-FU exposure. To achieve this, both HCT-C18 (TS+) and HCT-C18 (TS-) cells were treated with 10 μM 5-FU for 4 hrs and 24 hrs. Figures 4 and 6 listed genes with known functions based on One-way ANOVA clustering analysis from steady state total mRNA profiling from each cell line, respectively. A clear and unique expression pattern revealed a dynamic response to 5-FU treatment (Figure 4). There are 46 genes that were proved to be significant factors for acute 5-FU treatment (Genes listed in Additional file 3). They include CXCR4, FLJ23311, RSP26, AKAP12, ORC6L, PRPS1, DDX15, DKK4, and EIF4E. DKK4 functions as an agonist in the Wnt signaling pathway to stimulate cell proliferation during brain development [30]. However, little is known about the function of DKK4 in cancer. This is the first report to show that DKK4 may play an important role in colorectal cancer possibly via the Wnt signaling pathway [31]. eIF4E, one of the key translational initiation factors, has been shown to play key roles in cell cycle control and is an important marker for determining chemosensitivity [32,33]. We speculate that the acute induction of eIF4E expression by 5-FU may be contributing to the resistance mechanism. Following 24 hr 5-FU exposure, nearly all of the acutely up-regulated genes had returned to baseline levels. However, additional genes such as SPRR1A, DDB2, and CDKN1A were only up-regulated after 24 hrs. These differential gene expression profiles suggest that single time point microarray data from clinical samples should be viewed with caution and not over-interpreted.

Interestingly, we discovered an additional 67 genes associated with for 5-FU treatment based on profiling of polysome associated mRNA transcripts (Figure 5). The induction of p53 will contribute a significant part for cell cycle arrest and apoptosis based on previous reports [34,35]. In addition, we also found genes such as, G-protein coupled receptor 132 (G2A), colon cancer antigen 33 (SDCCAG33), TSC2, and p34 (SEI1), were perturbed with 5-FU exposure. SEI1 is a CKD4-binding protein to regulate CDK4 activity [36] and was up-regulated following 5-FU exposure. G2A and SDCCAG33 were acutely up-regulated by 5-FU treatment whereas TSC2 was a delayed response gene affected by 5-FU. It has been recently shown that TSC2 is regulated at the translational level by hypoxia [37]. TSC2 is also involved in mTOR signaling pathway to regulate cell growth, proliferation, and cell death [38]. mTOR is one of the key pathways for translational regulation via eIF4E [39]. These gene expression profiles may be helpful in elucidating additional resistance mechanisms related to 5-FU treatment.

We also determined the marker genes that were associated with TS independent cytotoxicity using HCT-C18 (TS-) cells treated with 5-FU in the presence of 10 μM thymidine. The main cytotoxicity caused by 5-FU was the inhibition of TS to block the sole *de novo *synthesis of thymidylate, an essential precursor for DNA biosynthesis. However, in the presence of 10 μM thymidine, the cytotoxicity caused by 5-FU in HCT-C18 (TS-) cells was mainly by the direct incorporation of 5-FU metabolite into DNA and RNA. The genes involved in TS independent cellular toxicity have not been systematically investigated in the past. In this study, we discovered a number of genes that were associated with TS independent cytotoxicity of 5-FU. Genes involved in DNA replication (NFIC, CDC45L, TREX1, UBE1), DNA repair (TREX1, BTG2, XPC), DNA metabolism (TK1), RNA catabolism (ELAVL1), RNA processing (U3-55K), cell signaling (DDK2, CAPRI, TFAP2C), and apoptosis (NFKB1A, TRAF4) are on this list (Figure 6 and Additional file 5). It's not surprising to see these genes on the list because direct incorporation of 5-FU metabolite to RNA and DNA is the major part of cytotoxicity via TS independent mechanism [40,41]. It has been shown that incorporation of 5-FU into RNA affects pre-mRNA splicing process [42]. This study provides the molecular targets and regulatory network that are potentially responsible for future enhancement of such cellular toxicity to tumors.

Genes associated with TS independent cytotoxicity caused by 5-FU were listed in Table 1. This list includes genes such as RRM2, SEI1, GPIBB, DDB2, and CDKN1A. A recent report showed that RRM2 (ribonucleotide reductase subunit M) was regulated at the translational level by the upstream AUGs [43]. This finding provides further validation to our approach as a systematic discovery platform for novel translational regulated genes. It has been shown recently that decreasing RRM2 level in HCT-116 (p53-/-) cells sensitize cells to DNA damaging agents and ribonucleotide reductase inhibitors [44]. It is clear that multiple genes will be responsible for determining the sensitivity in response to 5-FU other than TS itself [45].

These marker genes are important for us to understand the complex network regulated by TS and 5-FU. To go a step further, we also attempted to analyze them in the context of the regulatory network. As an example, we try to put these genes into a biological context via analyzing the cell cycle related genes affected by TS over-expression using pathway analysis, and the results are shown in Figure 7. It's quite clear that TS over-expression decreases p53 level, thereby affecting its downstream genes such as p21 and the rest of the cell cycle related genes. This approach also helps to visualize the inter-relationship between TS and cell cycle related genes. We believe this is a starting point to show the power of systems biology approach for dissecting the mechanism and network of gene regulation. Preliminary results using colon cancer patient samples demonstrated that some of these genes can be used as predictors for 5-FU based therapy (manuscript in preparation).

## Conclusion

In conclusion, the studies presented here provide a comprehensive expression profile of transcriptional and post-transcriptional levels affected by over-expression of TS protein and 5-FU treatment. This work expands our current understanding of the complex networks regulated by TS protein. In addition, our study also discovered candidate genes associated with TS independent cellular cytotoxicity. In particular, the newly discovered post-transcriptionally regulated genes by TS and 5-FU will be good candidates for further investigating the molecular and cellular mechanism of such regulation. This study further demonstrates the importance of understanding translational control in a global context in response to genotoxic stresses such as exposure to chemotherapeutic agents.

## Methods

### Cell culture

The human colon cancer cell lines, HCT-C18 (TS-) and HCT-C18 (TS+), have been previously described [16,20]. HCT-C18 (TS-) cells were maintained in 75-cm^2 ^plastic tissue culture flasks with growth medium consisting of RPMI 1640 medium containing 10% fetal bovine serum and supplemented with 10 μM thymidine (Sigma, MO) at 37°C and 5% CO_2_. The HCT-C18 (TS+) cells were growing in the same medium without thymidine.

### Western immunoblot analysis

Equal amounts of protein (25 μg) from each cell line were resolved by SDS/PAGE on 10% gels by the method of Laemmli [46]. Proteins were probed with mouse anti-TS monoclonal antibody (1:5,000 dilution) (Zymed Laboratories, CA), or anti-p53 mouse monoclonal antibody (1:1,000 dilution) (Santa Cruz, CA), anti-α-tubulin mouse monoclonal antibody (1:1,000 dilution) (Santa Cruz, CA) followed by secondary antibody (Bio-Rad, CA). Proteins were visualized with a chemiluminescence detection system using the Super Signal substrate (Pierce, IL).

### Isolation of polysome associated mRNA transcripts

For preparation of cytoplasmic extracts, cells from three 175 cm^2 ^tissue culture plates (60% confluency) were treated with 100 μg/ml cycloheximide (Sigma, MO) for 5 min at 37°C, washed with ice-cold phosphate-buffered saline containing cycloheximide (100 μg/ml) and harvested by scraping [47]. The fresh cells were pelleted by centrifugation, swollen for 2 min in 375 μl of low salt buffer (LSB) (20 mM Tris pH 7.5, 10 mM NaCl and 3 mM MgCl_2_) containing 1 mM dithiothreitol and 50 U recombinant RNasin (Promega, WI) and lysed by addition of 125 μl of lysis buffer (1× LSB containing 0.2 M sucrose and 1.2% Triton X-100) (Sigma) followed by vortexing. The nuclei were pelleted by centrifugation in a microcentrifuge at 13,000 rpm for 2 min. The supernatant (cytoplasmic extract) was transferred to a new 1.5 ml tube on ice. Cytoplasmic extracts were carefully layered over 0.5–1.5 M linear sucrose gradients (in LSB) and centrifuged at 45,000 rpm in a Beckman SW40 rotor for 90 min at 4°C. Gradients were fractionated using a pipette and the absorbance at 260 nm was measured for each fraction by UV spectrometry. Fractions 6 to 13 containing polysomes based on the positive absorbance profiles at 260 nm were pooled for RNA isolation.

### Sample preparation, array hybridization and gene expression analysis

CodeLink UniSet Human 20 K Oligo Bioarray (Amersham Biosciences, NJ), containing approximately 20,289 genes probes, was used to generate gene expression profiles of both steady state total mRNAs and actively translated mRNAs isolated from control HCT-C18 (TS-) and HCT-C18 (TS+) cells and cells treated with 10 μM 5-FU (n = 3). Steady state total mRNAs from untreated HCT-C18 TS (-) and HCT-C18 TS (+) cells or after treatment with 10 μM 5FU for 4 and 24 hrs were isolated using Trizol Reagent (n = 3) (Invitrogen, CA). The corresponding polysomal fractions from each sample were pooled together and actively translated mRNAs were isolated using Trizol LS Reagent (Invitrogen, CA). All reagents were provided in the CodeLink expression assay kit (Amersham Biosciences, NJ), except where noted. cRNA synthesis was performed as per manufacturer's instructions. Using 2 μg of total RNA, first-strand cDNA was generated by reverse transcriptase and a T7 primer. Subsequently, second-strand cDNA was produced using DNA polymerase I and RNase H. The resulting double-stranded cDNA was purified on a QIAquick column (Qiagen, CA) and cRNA was generated via an in vitro transcription reaction using T7 RNA polymerase and biotin-11-UTP (Perkin-Elmer, MA) at 37°C for 14 hrs. cRNA was purified on an RNeasy column (Qiagen, CA), quantified by UV spectrophotometry, and 10 μg biotin-labelled cRNA was then fragmented by heating at 94°C for 20 min in the presence of magnesium buffer. The fragmented cRNA was hybridized overnight at 37°C in hybridization buffer to a UniSet Human 20 K Bioarray in a shaking incubator at 300 rpm.

After hybridization, the arrays were washed in 0.75× TNT buffer [1× TNT: 0.1 M Tris-HCl (pH 7.6), 0.15 M NaCl, and 0.05% Tween 20] at 46°C for 1 hr followed by incubation with Cy5-streptavadin (Amersham Biosciences, NJ) at room temperature for 30 minutes in the dark. Arrays were then washed in 1× TNT four times for 5 min each. The slides were then dried by centrifugation and kept in the dark until scanning. Images were captured on an Axon GenePix 4200 A scanner. The resulting image was quantified and the intensity of each spot divided by the median spot intensity to provide a scaled and comparable number across multiple arrays. Bacterial spots provide both positive and negative controls. After dot grid and QC, CodeLink software generates export files for analysis by GeneSpring software 7.2 (Aglient, CA).

Gene expression analysis was carried out on GeneSpring software version 7.2, which allows multifilter comparisons using data from different experiments to perform the normalization, generation of restriction lists and the functional classification of the differentially expressed genes. Under Cross-Gene Error Model, normalization was applied in two steps: (a) "per chip normalization" in which each measurement was divided by the 50th percentile of all measurements in its array; and (b) "per gene normalization" in which all the samples were normalized against the specific samples (controls). Then data were filtered by flags and 4-fold change. The expression profiles of the different groups were compared using One-way ANOVA with cut-off p < 0.05. Comparisons of gene lists across different groups were performed using Venn Diagrams and clustering was performed with the Condition Tree algorithm. In addition, the Gene Ontology groupings and Gen Maps 2.0 program were used in conjunction with GeneSpring to identify pathways and functional groups of genes.

## Authors' contributions

YX performed the tissue culture and microarray gene expression profiling, data analysis and confirmation. GN performed the Western immunoblot analysis and assistance to the project. JS provided the critical review and scientific input of the manuscript. EC provided the cell lines and the critical scientific design and review of the project. JJ conceived, designed and led the project. All authors read and approved the manuscript.

## Supplementary Material

Additional File 1 **Gene expression affected by TS over-expression based on profiling steady state mRNAs in HCT-C18 (TS-) and HCT-C18 (TS+) cells. **This file contains the global gene expression profile of HCT-C18 (TS-) and HCT-C18 (TS+) cells based on steady state total mRNA expression using human high density CodeLink oligo array (20 K). Over 38 genes were changed in their expression in response to TS expression (n = 3, p < 0.05 and 4-fold cut-off). Click here for file

Additional File 2 **Gene expression affected by TS over-expression based on profiling polysome associated mRNAs in HCT-C18 (TS-) and HCT-C18 (TS+) cells. **This file contains the gene list of potential novel genes controlled at the post-transcriptionally by TS protein. Polysome associated mRNAs from both HCT-C18 (TS-) and HCT-C18 (TS+) cells were isolated and expression analysis was performed using human high density CodeLink oligo array (20 K). Over 149 genes were effected in TS overexpressing HCT-C18 (TS+) cells (n = 3, p < 0.05 with 4-fold cut-off). Click here for file

Additional File 3 **Effect of 5-FU treatment on steady state mRNAs expression in HCT-C18 (TS+) cells. **This file contains a gene list associated with both acute and delayed response genes following 5-FU treatment in HCT-C18 (TS+) cells by gene expression profiling analysis using steady state total mRNAs isolated from control and 5-FU treated samples at 4 hrs and 24 hrs time points. The expression analysis reveals that over 46 genes were affected by 5-FU treatment by One-way ANOVA analysis (n = 3, p < 0.05 with 4-fold cut-off). The clustering analysis is shown in Figure 4. Click here for file

Additional File 4 **Effect of 5-FU treatment on polysome associated mRNAs expression in HCT-C18 (TS+) cells. **This file contains both acute and delayed response genes that were affected at post-transcriptional level by 5-FU treatment. Polysome associated mRNAs were isolated from control, and 5-FU treated samples at 4 hrs and 24 hrs. Gene expression analysis via microarray revealed that over 67 genes were affected in response to 5-FU (n = 3, p < 0.05 with 4-fold cut off). The clustering analyses are shown in Figure 5. Click here for file

Additional File 5 **Effect of 5-FU treatment on steady state mRNAs expression in HCT-C18 (TS-) cells. **This file contains gene list based on gene expression profiles generated from these samples. These genes are mainly associated with TS independent toxicity in response to 5-FU by direct incorporation to RNA and DNA. The clustering analysis is shown in Figure 6. This file contains a gene list of 185 genes (n = 3, p < 0.05 with 4-fold cut-off) affected by 5-FU exposure in HCT-C18 (TS-) cells in the presence of thymidine. Click here for file

## Figures and Tables

**Table 1:** Overlapping genes between steady state and polysome associated mRNAs affected by TS overexpression.'

**Genebank accession**	**Gene ID**	**Fold change**		**Biological function**
			
		**Total**	**Polysome**	
**Increased genes**				
NM_002192	INHBA	+21.81	+33.02	Cell cycle arrest; cell differentiation; signal transduction; cell-cell signaling;
NM_000930	PLAT	+11.93	+6.73	Blood coagulation; protein modification; proteolysis and peptidolysis
AB032261	SCD	+5.48	+6.62	Fatty acid biosynthesis
**Decreased genes**				
NM_001442	FABP4	-100.00	-100.00	Transport
NM_002153	HSD17B2	-54.95	-100.00	Estrogen biosynthesis; metabolism
NM_033292	CASP1;COP	-22.22	-11.68	Regulation of I-κB kinase/NF-κB cascade; apoptosis; signal transduction
NM_003641	IFITM1	-18.08	-23.58	Signal transduction; immune response; cell proliferation and cycle
NM_002274	KRT13	-7.81	-11.90	Epidermis development
NM_002638	PI3	-5.75	-10.41	Copulation

**Table 2:** 2

**Genebank accession**	**Gene ID**	**P value **		**Biological function**
			
		**TS+**	**TS-**	
S73288	SPRR1A	0.0073	0.0078	Epidermis development
NM_001034	RRM2	0.0143	0.0133	DNA replication; deoxyribonucleoside diphosphate metabolism
NM_005554	KRT6A, B, C	0.0168	0.0030	Ectoderm development
NM_003125	SPRR1B	0.0211	0.0014	Epidermis development
NM_000407	GP1BB	0.0229	0.0094	Blood coagulation; cell adhesion; cell surface receptor linked signal transduction
NM_000389	CDKN1A	0.0265	0.0137	Cell cycle arrest; apoptosis;cell proliferation; regulation of cyclin dependent protein kinase activity
NM_013376	SEI1	0.0274	0.0477	Cell proliferation; regulation of cyclin dependent protein kinase activity; transcription, DNA-dependent
NM_000422	KRT17	0.0278	0.0044	Epidermis development
NM_002658	PLAU	0.0362	0.0019	Blood coagulation; cell growth and/or maintenance; chemotaxis
NM_000107	DDB2	0.0441	0.0049	Nucleotide-excision repair
NM_006142	SFN	0.0482	0.0115	Cell proliferation; negative regulation of protein kinase activity; regulation of cell cycle; signal transduction

Overlapping genes between HCT-C18 (TS+) and HCT-C18 (TS-) cells affected by 5-FU treatment.
