# Supplementary material for: Multi-level gene expression profiles affected by thymidylate synthase and 5-fluorouracil in colon cancer
Source: BMC Genomics. 2006 Apr 3;7:68. doi: 10.1186/1471-2164-7-68 (PMC1448211; doi:10.1186/1471-2164-7-68)
Supplement: Additional File 1 — Gene expression affected by TS over-expression based on profiling steady state mRNAs in HCT-C18 (TS-) and HCT-C18 (TS+) cells. This file contains the global gene expression profile of HCT-C18 (TS-) and HCT-C18 (TS+) cells based on steady state total mRNA expression using human high density CodeLink oligo array (20 K). Over 38 genes were changed in their expression in response to TS expression (n = 3, p < 0.05 and 4-fold cut-off). [file 1471-2164-7-68-S1.doc]

# Additional file 1

### Gene expression affected by TS overexpression based on profiling steady state mRNAs in HCT-C18 (TS-) and HCT-C18 (TS+) cells (n=3, p<0.05 with 4-fold cut-off). (38 genes)

| **Genebank access number** | **Genes ID** | **Fold change** | **Biological function** |
| --- | --- | --- | --- |
| **Increased Genes** |  |  |  |
| NM_014483 | RBMS3 | +28.71 | Nuclear mrna splicing, via spliceosome |
| NM_002192 | INHBA | +21.81 | Cell cycle arrest; cell differentiation; cell growth and/or maintenance; cell surface receptor linked signal transduction; cell-cell signaling; defense response; erythrocyte differentiation; hemoglobin biosynthesis; induction of apoptosis; mesoderm development; negative regulation of B-cell differentiation; negative regulation of cell cycle; negative regulation of follicle-stimulating hormone secretion; negative regulation of interferon-gamma biosynthesis; negative regulation of macrophage differentiation; negative regulation of phosphorylation; neurogenesis; ovarian follicle development; positive regulation of follicle-stimulating hormone secretion; response to external stimulus; skeletal development |
| NM_001353 | AKR1C1;AKR1C2 | +20.64 | Xenobiotic metabolism |
| NM_005954 | MT3 | +15.96 | Cell proliferation; electron transport; metal ion homeostasis; negative regulation of cell growth; negative regulation of dendrite morphogenesis; removal of superoxide radicals; response to hypoxia |
| NM_017855 | FLJ20513 | +14.06 |  |
| NM_014716 | CENTB1 | +13.21 | Intracellular signaling cascade |
| NM_002847 | PTPRN2 | +12.70 | Protein amino acid dephosphorylation |
| NM_000930 | PLAT | +11.93 | Blood coagulation; protein modification; proteolysis and peptidolysis |
| AL079279 |  | +11.59 |  |
| AB006627 | ASTN | +11.46 | Cell adhesion; cell migration; neuronal cell adhesion |
| BC006831 | H19 | +9.80 |  |
| NM_000055 | BCHE | +7.77 | Cocaine metabolism |
| NM_001988 | EVPL | +7.49 | Epidermis development |
| X69141 | FDFT1 | +5.84 | Biosynthesis; cholesterol biosynthesis; isoprenoid biosynthesis; steroid biosynthesis |
| BE221303 |  | +5.67 |  |
| AB032261 | SCD | +5.48 | Fatty acid biosynthesis |
| **Decreased Genes** |  |  |  |
| NM_001442 | FABP4 | -100.00 | Transport |
| NM_002153 | HSD17B2 | -54.95 | Estrogen biosynthesis; metabolism |
| AL137343 | NSE1 | -36.36 |  |
| NM_006183 | NTS | -32.57 | Signal transduction |
| NM_033292 | CASP1;COP | -22.22 | Positive regulation of I-kappab kinase/NF-kappab cascade; proteolysis and peptidolysis; regulation of apoptosis; signal transduction |
| NM_003641 | IFITM1 | -18.08 | Cell surface receptor linked signal transduction; immune response; negative regulation of cell proliferation; regulation of cell cycle; response to biotic stimulus |
| S60415 | CACNB2 | -13.51 | Calcium ion transport; neuromuscular junction development |
| NM_000963 | PTGS2 | -12.58 | Cell motility; cyclooxygenase pathway; physiological process; regulation of inflammatory response |
| NM_006408 | AGR2 | -9.52 |  |
| NM_032024 | CDA017 | -8.06 |  |
| NM_002274 | KRT13 | -7.81 | Epidermis development |
| NM_032622 | LNX | -7.14 | Protein ubiquitination |
| AY069977 | LOC155465 | -6.99 |  |
| NM_000700 | ANXA1 | -6.33 | Cell motility; cell surface receptor linked signal transduction; inflammatory response; lipid metabolism |
| AL163300 |  | -5.75 |  |
| NM_002638 | PI3 | -5.75 | Copulation |
| NM_012449 | STEAP | -5.21 |  |
| NM_000716 | C4BPB | -5.15 | Blood coagulation; complement activation, classical pathway |
| NM_032638 | MGC2306 | -4.85 | Cell growth and/or maintenance; regulation of transcription, DNA-dependent; transcription from Pol II promoter |
| AB051510 | DLC1 | -4.57 | Cytoskeleton organization and biogenesis; negative regulation of cell growth; regulation of cell adhesion |
| NM_006472 | TXNIP | -4.29 | Biological_process unknown |
| NM_003064 | SLPI | -4.03 |  |

# 
