# Supplementary material for: Multi-level gene expression profiles affected by thymidylate synthase and 5-fluorouracil in colon cancer
Source: BMC Genomics. 2006 Apr 3;7:68. doi: 10.1186/1471-2164-7-68 (PMC1448211; doi:10.1186/1471-2164-7-68)
Supplement: Additional File 2 — Gene expression affected by TS over-expression based on profiling polysome associated mRNAs in HCT-C18 (TS-) and HCT-C18 (TS+) cells. This file contains the gene list of potential novel genes controlled at the post-transcriptionally by TS protein. Polysome associated mRNAs from both HCT-C18 (TS-) and HCT-C18 (TS+) cells were isolated and expression analysis was performed using human high density CodeLink oligo array (20 K). Over 149 genes were effected in TS overexpressing HCT-C18 (TS+) cells (n = 3, p < 0.05 with 4-fold cut-off). [file 1471-2164-7-68-S2.doc]

# Additional file 2

### Gene expression affected by TS overexpression based on profiling polysome associated mRNAs in HCT-C18 (TS-) and HCT-C18 (TS+) cells (n=3, p<0.05 with 4-fold cut-off). (149 genes)

| **Genebank access number** | **Genes ID** | **Fold change** | **Biological function** |
| --- | --- | --- | --- |
| **Increased genes** |  |  |  |
| AF123539 | HTCD37 | +2,003.00 | Biological_process unknown |
| NM_017910 | FLJ20628 | +1,645.00 |  |
| NM_017855 | FLJ20513 | +51.83 |  |
| AK022251 | FLJ10989 | +42.11 | Protein biosynthesis |
| AA102395 |  | +35.72 |  |
| NM_002192 | INHBA | +33.02 | Cell cycle arrest; cell differentiation; cell growth and/or maintenance; cell surface receptor linked signal transduction; cell-cell signaling; defense response; erythrocyte differentiation; hemoglobin biosynthesis; induction of apoptosis; mesoderm development; negative regulation of B-cell differentiation; negative regulation of cell cycle; negative regulation of follicle-stimulating hormone secretion; negative regulation of interferon-gamma biosynthesis; negative regulation of macrophage differentiation; negative regulation of phosphorylation; neurogenesis; ovarian follicle development; positive regulation of follicle-stimulating hormone secretion; response to external stimulus; skeletal development |
| AJ420510 | XAP135 | +23.40 | Regulation of transcription, DNA-dependent |
| NM_000466 | PEX1 | +21.04 | Peroxisome organization and biogenesis; protein transport |
| NM_000318 | PXMP3 | +17.74 | Peroxisome organization and biogenesis |
| NM_014350 | GG2-1 | +17.26 | Anti-apoptosis |
| NM_002835 | PTPN12 | +12.78 | Protein amino acid dephosphorylation |
| BC020584 | DKFZP564D172 | +12.53 |  |
| NM_003136 | SRP54 | +12.34 | SRP-dependent cotranslational membrane targeting; protein targeting |
| NM_017827 | FLJ20450 | +11.19 | Protein biosynthesis; seryl-trna aminoacylation |
| NM_005038 | PPID | +10.48 | Protein folding |
| AB050468 | LIG1 | +10.41 |  |
| AB028963 | KIAA1040 | +10.14 |  |
| NM_013263 | BRD7 | +10.06 |  |
| NM_020215 | DKFZp761F2014 | +10.03 |  |
| NM_000888 | ITGB6 | +10.02 | Cell-matrix adhesion; integrin-mediated signaling pathway |
| NM_001682 | ATP2B1 | +9.94 | Calcium ion transport; cation transport; metabolism |
| NM_019005 | FLJ20323 | +9.58 |  |
| AF346509 | NFAT5 | +9.28 | Excretion; regulation of transcription, DNA-dependent; signal transduction; transcription from Pol II promoter |
| AF055030 | XAP135 | +9.23 |  |
| NM_004165 | RRAD | +9.18 | Small gtpase mediated signal transduction |
| BC006831 | H19 | +9.16 |  |
| BM741997 | SLC25A3 | +9.01 |  |
| AK055660 | MTPN | +8.92 |  |
| NM_000194 | HPRT1 | +8.91 | Behavior; nucleoside metabolism; purine ribonucleoside salvage |
| NM_004578 | RAB4A | +8.83 | Intracellular protein transport; small gtpase mediated signal transduction |
| NM_014584 | ERO1L | +8.79 | Chaperone cofactor dependent protein folding; electron transport |
| NM_001695 | ATP6C | +8.70 | ATP synthesis coupled proton transport; cell surface receptor linked signal transduction; development |
| NM_031311 | CPVL | +8.63 | Proteolysis and peptidolysis |
| AB037723 | ODZ4 | +8.51 |  |
| AL137441 | CGI-77 | +8.31 |  |
| BC000862 | LOC87178 | +8.27 | RNA processing |
| NM_001779 | CD58 | +8.12 | Antimicrobial humoral response (sensu Vertebrata); cell-cell adhesion |
| NM_017544 | NRF | +8.11 | Regulation of transcription, DNA-dependent |
| AK057634 | PLCB4 | +8.06 | Intracellular signaling cascade; lipid catabolism |
| NM_003819 | PABPC4 | +8.04 | RNA catabolism; RNA processing; blood coagulation; protein biosynthesis; response to pest/pathogen/parasite |
| AL049319 | C10orf22 | +7.91 |  |
| NM_004046 | ATP5A1 | +7.83 | ATP synthesis coupled proton transport |
| NM_016258 | HGRG8 | +7.81 |  |
| NM_032632 | MGC5378 | +7.76 | Mrna polyadenylation; transcription |
| NM_005228 | EGFR | +7.71 | Cell proliferation; electron transport; epidermal growth factor receptor signaling pathway; protein amino acid phosphorylation |
| NM_004531 | MOCS2 | +7.71 | Mo-molybdopterin cofactor biosynthesis; sulfur metabolism |
| NM_003299 | TRA1 | +7.67 | Protein folding; response to stress |
| AB037745 | KIAA1324 | +7.63 |  |
| NM_014890 | DOC1 | +7.60 | Biological_process unknown |
| NM_138794 | LOC127018 | +7.57 |  |
| AB040893 | KIAA1460 | +7.53 | Nuclear mrna splicing, via spliceosome |
| NM_003400 | XPO1 | +7.47 | Protein transport; protein-nucleus import, docking |
| NM_003980 | MAP7 | +7.44 | Establishment and/or maintenance of cell polarity; microtubule cytoskeleton organization and biogenesis |
| NM_006417 | MTAP44 | +7.19 | Response to virus |
| NM_016229 | LOC51700 | +7.08 | Electron transport |
| NM_001889 | CRYZ | +6.98 | Visual perception |
| NM_005536 | IMPA1 | +6.93 | Carbohydrate metabolism; phosphate metabolism; phosphatidylinositol biosynthesis; signal transduction |
| NM_023012 | FLJ11021 | +6.92 |  |
| NM_001527 | HDAC2 | +6.92 |  |
| NM_003129 | SQLE | +6.87 | Aromatic compound metabolism; electron transport; sterol biosynthesis |
| NM_012244 | SLC7A8 | +6.81 | Amino acid metabolism; amino acid transport; protein complex assembly |
| NM_002431 | MNAT1 | +6.80 | DNA repair; cell cycle; protein complex assembly; regulation of cyclin dependent protein kinase activity; regulation of transcription from Pol II promoter |
| NM_000930 | PLAT | +6.73 | Blood coagulation; protein modification; proteolysis and peptidolysis |
| NM_005111 | CRYZL1 | +6.71 |  |
| NM_012096 | APPL | +6.69 | Cell proliferation; signal transduction |
| NM_002806 | PSMC6 | +6.68 | Protein catabolism |
| AB032261 | SCD | +6.62 | Fatty acid biosynthesis |
| AL157449 | PPP1R9B | +6.51 |  |
| AL110136 |  | +6.49 |  |
| NM_002358 | MAD2L1 | +6.48 | Cell cycle; mitosis; mitotic checkpoint |
| NM_006350 | FST | +6.46 | Development; negative regulation of follicle-stimulating hormone secretion |
| NM_005798 | RFP2 | +6.46 | Morphogenesis; negative regulation of cell cycle; positive regulation of I-kappab kinase/NF-kappab cascade |
| AI927931 | MPRP-1 | +6.39 |  |
| AB029020 | VDU1 | +6.29 | Protein deubiquitination; ubiquitin-dependent protein catabolism |
| AI888150 |  | +6.27 |  |
| NM_007213 | JM4 | +6.24 |  |
| NM_005358 | LMO7 | +6.15 | Protein ubiquitination |
| NM_022662 | MCPR | +6.10 | Cytokinesis; mitosis; regulation of cell cycle; ubiquitin cycle |
| NM_018657 | MYNN | +5.98 | Transcription, DNA-dependent |
| AB014597 | KIAA0697 | +5.98 | Mismatch repair |
| NM_014426 | SNX5 | +5.98 | Intracellular signaling cascade |
| NM_001425 | EMP3 | +5.97 | Cell death; cell growth; cell proliferation; development; negative regulation of cell proliferation |
| AK000822 | DKFZP564M182 | +5.96 | Protein biosynthesis |
| AF084943 | MINPP1 | +5.95 | Bone mineralization; polyphosphate metabolism |
| AI652868 |  | +5.94 |  |
| NM_006055 | LANCL1 | +5.94 | G-protein coupled receptor protein signaling pathway |
| NM_006731 | FCMD | +5.94 | Muscle development; neurogenesis |
| NM_021159 | RAP1GDS1 | +5.93 | Biological_process unknown |
| NM_006585 | CCT8 | +5.92 |  |
| BC035090 | KPNA3 | +5.84 | NLS-bearing substrate-nucleus import; intracellular protein transport; protein complex assembly |
| NM_016570 | LOC51290 | +5.84 |  |
| NM_013450 | BAZ2B | +5.81 | Regulation of transcription, DNA-dependent |
| AK027561 | FLJ10006 | +5.79 | Regulation of transcription, DNA-dependent |
| NM_004724 | ZW10 | +5.76 | Cell cycle; meiosis; mitosis; mitotic checkpoint; mitotic sister chromatid segregation; protein complex assembly; regulation of exit from mitosis; transport |
| NM_014763 | MRPL19 | +5.75 | Protein biosynthesis |
| NM_007265 | HSGT1 | +5.75 | Regulation of glycolysis; transcription from Pol II promoter |
| AL109695 | SLCO3A1 | +5.75 |  |
| AB037836 | KIAA1415 | +5.70 | Actin filament polymerization; intracellular signaling cascade; neutrophil activation; superoxide metabolism |
| NM_004628 | XPC | +5.64 | Nucleotide-excision repair |
| Y10183 | ALCAM | +5.63 | Antimicrobial humoral response (sensu Vertebrata); cell adhesion; signal transduction |
| NM_003139 | SRPR | +5.62 | SRP-dependent cotranslational membrane targeting; intracellular protein transport |
| NM_014448 | ARHGEF16 | +5.54 |  |
| AL080156 | DKFZP434J214 | +5.52 |  |
| AB033114 | ATIP1 | +5.50 |  |
| NM_006527 | SLBP | +5.50 | Histone mrna 3'-end processing |
| AL096751 | MPHOSPH9 | +5.49 | M phase of mitotic cell cycle; regulation of cell cycle |
| NM_133436 | ASNS | +5.48 | Asparagine biosynthesis; glutamine metabolism |
| NM_006660 | CLPX | +5.43 | Protein folding; protein transport |
| BC015050 | OIP5 | +5.37 | Cell communication |
| NM_014313 | SMP1 | +5.33 |  |
| NM_000126 | ETFA | +5.32 | Electron transport |
| NM_006214 | PHYH | +5.30 | Lipid metabolism; neurogenesis; perception of sound; visual perception |
| NM_025238 | BTBD1 | +5.30 | Biological_process unknown |
| AK057565 | FNBP2 | +5.22 |  |
| NM_003800 | RNGTT | +5.21 | Mrna capping; protein amino acid dephosphorylation |
| NM_004483 | GCSH | +5.18 | Glycine catabolism |
| AK001853 |  | +5.16 |  |
| NM_018256 | WDR12 | +5.16 |  |
| NM_006023 | D123 | +5.15 | Cell cycle arrest; positive regulation of cell proliferation |
| NM_004496 | HNF3A | +5.15 | Regulation of transcription, DNA-dependent |
| NM_002710 | PPP1CC | +5.11 | Cytokinesis; glycogen metabolism |
| NM_016217 | LOC51696 | +5.10 | Development; regulation of cell cycle; respiratory tube development |
| NM_000901 | NR3C2 | +5.10 | Excretion; regulation of transcription, DNA-dependent; signal transduction; sodium ion homeostasis |
| NM_005901 | MADH2 | +5.07 | Regulation of transcription, DNA-dependent; signal transduction |
| NM_007208 | MRPL3 | +5.04 | Protein biosynthesis |
| NM_015686 | TED | +4.99 | Development |
| NM_001688 | ATP5F1 | +4.97 | ATP synthesis coupled proton transport |
| NM_001539 | DNAJA1 | +4.96 | Protein folding |
| **Decreased genes** |  |  |  |
| NM_001442 | FABP4 | -100.00 | Transport |
| NM_002153 | HSD17B2 | -100.00 | Estrogen biosynthesis; metabolism |
| AL137343 | NSE1 | -71.94 |  |
| NM_005742 | P5 | -27.78 | Electron transport; protein folding |
| NM_003641 | IFITM1 | -23.58 | Cell surface receptor linked signal transduction; immune response; negative regulation of cell proliferation; regulation of cell cycle; response to biotic stimulus |
| NM_024565 | FLJ14166 | -15.63 | Cell cycle; cytokinesis; regulation of cell cycle |
| NM_002274 | KRT13 | -11.90 | Epidermis development |
| NM_033292 | CASP1;COP | -11.68 | Positive regulation of I-kappab kinase/NF-kappab cascade; proteolysis and peptidolysis; regulation of apoptosis; signal transduction |
| NM_002638 | PI3 | -10.41 | Copulation |
| NM_006408 | AGR2 | -9.17 |  |
| NM_013376 | SEI1 | -7.19 | Positive regulation of cell proliferation; regulation of cyclin dependent protein kinase activity; regulation of transcription, DNA-dependent |
| NM_005978 | S100A2 | -6.94 | Biological_process unknown |
| NM_001188 | BAK1 | -6.54 | Apoptotic mitochondrial changes; induction of apoptosis; regulation of apoptosis |
| NM_006149 | LGALS4 | -6.29 | Cell adhesion; heterophilic cell adhesion |
| NM_003064 | SLPI | -6.10 |  |
| U88834 | LOC92558 | -5.78 |  |
| NM_023944 | CYP4F12 | -5.71 | Electron transport |
| NM_002105 | H2AFX | -5.43 | Chromosome organization and biogenesis (sensu Eukarya); nucleosome assembly |
| NM_005837 | RPP20 | -5.35 | Trna processing |
| NM_000389 | CDKN1A | -5.32 | Cell cycle arrest; induction of apoptosis by intracellular signals; negative regulation of cell proliferation; regulation of cyclin dependent protein kinase activity |
| NM_032024 | CDA017 | -5.05 |  |
| NM_000546 | TP53 | -4.50 | Tumor suppressor; cell cycle arrest; apoptosis |

### 
