# Supplementary material for: Multi-level gene expression profiles affected by thymidylate synthase and 5-fluorouracil in colon cancer
Source: BMC Genomics. 2006 Apr 3;7:68. doi: 10.1186/1471-2164-7-68 (PMC1448211; doi:10.1186/1471-2164-7-68)
Supplement: Additional File 3 — Effect of 5-FU treatment on steady state mRNAs expression in HCT-C18 (TS+) cells. This file contains a gene list associated with both acute and delayed response genes following 5-FU treatment in HCT-C18 (TS+) cells by gene expression profiling analysis using steady state total mRNAs isolated from control and 5-FU treated samples at 4 hrs and 24 hrs time points. The expression analysis reveals that over 46 genes were affected by 5-FU treatment by One-way ANOVA analysis (n = 3, p < 0.05 with 4-fold cut-off). The clustering analysis is shown in Figure 4. [file 1471-2164-7-68-S3.doc]

# Additional file 3

### Effect of 5-FU treatment on steady state mRNAs expression in HCT-C18 (TS+) cells. (46 genes)

| **Genebank access number** | **Gene ID** | **P value** | **Biological function** |
| --- | --- | --- | --- |
| AA147817 |  | 0.0046 |  |
| S73288 | SPRR1A | 0.0073 | Epidermis development |
| NM_001034 | RRM2 | 0.0143 | DNA replication; deoxyribonucleoside diphosphate metabolism |
| NM_138611 | HCC4 | 0.0145 |  |
| NM_005416 | SPRR3 | 0.0148 |  |
| NM_003467 | CXCR4 | 0.0154 | G-protein coupled receptor protein signaling pathway; activation of MAPK; apoptosis; chemotaxis; cytosolic calcium ion concentration elevation; immune response; inflammatory response; neurogenesis; response to virus |
| NM_024680 | FLJ23311 | 0.0154 | Regulation of cell cycle; regulation of transcription, DNA-dependent |
| NM_005978 | S100A2 | 0.0157 | Biological_process unknown |
| NM_005554 | KRT6A;KRT6B;KRT6C | 0.0168 | Ectoderm development |
| NM_000716 | C4BPB | 0.0176 | Blood coagulation; complement activation, classical pathway |
| NM_003125 | SPRR1B | 0.0211 | Epidermis development |
| NM_002153 | HSD17B2 | 0.0219 | Estrogen biosynthesis; metabolism |
| AL163262 |  | 0.0229 |  |
| NM_000407 | GP1BB | 0.0229 | Blood coagulation; cell adhesion; cell surface receptor linked signal transduction |
| BC002604 | RPS26 | 0.0249 | Protein biosynthesis |
| NM_005100 | AKAP12 | 0.0249 | G-protein coupled receptor protein signaling pathway; protein targeting; signal transduction |
| NM_000389 | CDKN1A | 0.0265 | Cell cycle arrest; induction of apoptosis by intracellular signals; negative regulation of cell proliferation; regulation of cyclin dependent protein kinase activity |
| NM_003548 | H4F2 | 0.0267 |  |
| NM_013376 | SEI1 | 0.0274 | Positive regulation of cell proliferation; regulation of cyclin dependent protein kinase activity; regulation of transcription, DNA-dependent |
| NM_000422 | KRT17 | 0.0278 | Epidermis development |
| NM_002105 | H2AFX | 0.0348 | Chromosome organization and biogenesis (sensu Eukarya); nucleosome assembly |
| NM_002658 | PLAU | 0.0362 | Blood coagulation; cell growth and/or maintenance; chemotaxis; negative regulation of blood coagulation; proteolysis and peptidolysis; signal transduction |
| AI034351 |  | 0.0375 |  |
| NM_014321 | ORC6L | 0.0380 | DNA replication |
| NM_014471 | SPINK4 | 0.0382 |  |
| AL137763 | LOC57822 | 0.0386 |  |
| BQ670872 | FAM20C | 0.0395 |  |
| NM_018518 | MCM10 | 0.0399 |  |
| D84276 | CD38 | 0.0418 | Energy pathways; induction of apoptosis by extracellular signals; signal transduction |
| NM_002764 | PRPS1 | 0.0425 | Nucleoside metabolism; nucleotide biosynthesis; ribonucleoside monophosphate biosynthesis |
| AL834372 | DKFZp762F0713 | 0.0441 |  |
| NM_000107 | DDB2 | 0.0441 | Nucleotide-excision repair |
| NM_000584 | IL8 | 0.0442 | G-protein coupled receptor protein signaling pathway; angiogenesis; calcium-mediated signaling; cell cycle arrest; cell motility; cell-cell signaling; chemotaxis; immune response; induction of positive chemotaxis; intracellular signaling cascade; negative regulation of cell proliferation; neutrophil activation; neutrophil chemotaxis; regulation of cell adhesion; regulation of retroviral genome replication |
| NM_006319 | CDIPT | 0.0443 | Phospholipid biosynthesis |
| NM_001358 | DDX15 | 0.0448 | Nuclear mrna splicing, via spliceosome |
| AB007974 |  | 0.0459 |  |
| NM_014420 | DKK4 | 0.0461 | Wnt receptor signaling pathway; development; negative regulation of Wnt receptor signaling pathway |
| NM_020151 | STARD7 | 0.0468 |  |
| NM_001968 | EIF4E | 0.0469 | Regulation of protein biosynthesis; regulation of translation; translational initiation |
| NM_001268 | CHC1L | 0.0471 |  |
| NM_016095 | LOC51659 | 0.0472 | DNA replication |
| NM_005492 | CST8 | 0.0473 |  |
| NM_014874 | MFN2 | 0.0476 |  |
| NM_015474 | SAMHD1 | 0.0481 | Immune response; response to virus |
| NM_006142 | SFN | 0.0482 | Cell proliferation; negative regulation of protein kinase activity; regulation of cell cycle; signal transduction |
| NM_000336 | SCNN1B | 0.0496 | Excretion; ion transport; sodium ion transport |
