# Supplementary material for: Multi-level gene expression profiles affected by thymidylate synthase and 5-fluorouracil in colon cancer
Source: BMC Genomics. 2006 Apr 3;7:68. doi: 10.1186/1471-2164-7-68 (PMC1448211; doi:10.1186/1471-2164-7-68)
Supplement: Additional File 4 — Effect of 5-FU treatment on polysome associated mRNAs expression in HCT-C18 (TS+) cells. This file contains both acute and delayed response genes that were affected at post-transcriptional level by 5-FU treatment. Polysome associated mRNAs were isolated from control, and 5-FU treated samples at 4 hrs and 24 hrs. Gene expression analysis via microarray revealed that over 67 genes were affected in response to 5-FU (n = 3, p < 0.05 with 4-fold cut off). The clustering analyses are shown in Figure 5. [file 1471-2164-7-68-S4.doc]

# Additional file 4

Effect of 5-FU treatment on polysome associated mRNAs expression in HCT-C18 (TS+) cells. (67 genes)

| **Genebank access number** | **Gene ID** | **P value** | **Biological function** |
| --- | --- | --- | --- |
| NM_013345 | G2A | 0.0001 | G-protein coupled receptor protein signaling pathway |
| NM_005786 | SDCCAG33 | 0.0001 | Regulation of transcription, DNA-dependent |
| NM_021817 | BRAL1 | 0.0003 | Cell adhesion |
| BM477139 | VPS35 | 0.0036 |  |
| NM_001353 | AKR1C1;AKR1C2 | 0.0069 | Xenobiotic metabolism |
| NM_012219 | MRAS | 0.0114 | RAS protein signal transduction; actin cytoskeleton organization and biogenesis; development; muscle development |
| NM_020299 | AKR1B10 | 0.0122 | Biological_process unknown |
| NM_018187 | FLJ10707 | 0.0139 |  |
| AL137491 | LOC148696 | 0.0155 |  |
| NM_003739 | AKR1C3 | 0.0155 | Cell proliferation; lipid metabolism; prostaglandin metabolism |
| NM_000709 | BCKDHA | 0.0169 | Metabolism |
| NM_013262 | MIR | 0.0169 | Protein ubiquitination |
| AL157442 | GRINA | 0.0184 |  |
| NM_016316 | REV1L | 0.0218 | DNA repair; error-prone postreplication DNA repair; mutagenesis; response to UV |
| NM_013275 | LZ16 | 0.0219 |  |
| NM_030789 | HM13 | 0.0231 |  |
| NM_006947 | SRP72 | 0.0235 | Protein amino acid phosphorylation; signal transduction |
| AK094595 | UNC5H2 | 0.0236 | Signal transduction |
| AJ292190 | TAF3 | 0.0237 |  |
| NM_005859 | PURA | 0.0249 | DNA replication initiation; regulation of transcription, DNA-dependent |
| NM_000389 | CDKN1A | 0.0261 | Cell cycle arrest; induction of apoptosis by intracellular signals; negative regulation of cell proliferation; regulation of cyclin dependent protein kinase activity |
| NM_006142 | SFN | 0.0270 | Cell proliferation; negative regulation of protein kinase activity; regulation of cell cycle; signal transduction |
| NM_000548 | TSC2 | 0.0278 | Cell growth and/or maintenance; endocytosis; negative regulation of cell cycle; protein folding |
| NM_018306 | FLJ11036 | 0.0325 |  |
| NM_005919 | MEF2B | 0.0329 | Muscle development; regulation of transcription, DNA-dependent; transcription from Pol II promoter |
| BM453859 | HIC | 0.0334 |  |
| NM_001453 | FOXC1 | 0.0335 | Morphogenesis; regulation of transcription, DNA-dependent; visual perception |
| NM_002906 | RDX | 0.0335 | Cytoskeletal anchoring |
| NM_024319 | C1orf35 | 0.0335 |  |
| NM_005597 | NFIC | 0.0340 | DNA replication; regulation of transcription, DNA-dependent; transcription from Pol II promoter |
| NM_000872 | HTR7 | 0.0350 | G-protein signaling, coupled to cyclic nucleotide second messenger; circadian rhythm; circulation; synaptic transmission |
| NM_023006 | KLK15 | 0.0351 | Proteolysis and peptidolysis |
| NM_032753 | MGC15631 | 0.0351 | Regulation of transcription, DNA-dependent |
| NM_020345 | KBRAS1 | 0.0354 | I-kappab kinase/NF-kappab cascade; small gtpase mediated signal transduction |
| NM_014676 | PUM1 | 0.0356 | Mrna metabolism; regulation of translation |
| AK027033 | IREB2 | 0.0362 | Metabolism; negative regulation of translation |
| NM_017842 | FLJ20489 | 0.0372 |  |
| NM_001803 | CDW52 | 0.0378 |  |
| NM_014428 | TJP3 | 0.0379 |  |
| NM_005194 | CEBPB | 0.0381 | Acute-phase response; inflammatory response; regulation of transcription, DNA-dependent; transcription from Pol II promoter |
| AF209389 | CYP3A4 | 0.0384 | Electron transport; lipid metabolism; xenobiotic metabolism |
| NM_014925 | KIAA1002 | 0.0393 |  |
| NM_016653 | ZAK | 0.0398 | DNA damage response, signal transduction resulting in cell cycle arrest; activation of JUNK; activation of MAP/ERK kinase kinase; activation of MAPKK; cell death; cell differentiation; cell proliferation; protein amino acid phosphorylation; response to radiation; response to stress |
| NM_002500 | NEUROD1 | 0.0407 | Cell differentiation; neurogenesis; regulation of transcription, DNA-dependent |
| NM_000774 | CYP2F1 | 0.0411 | Electron transport |
| AL049365 | MGC50853 | 0.0412 |  |
| NM_000391 | CLN2 | 0.0413 | Lipid metabolism; neurogenesis; proteolysis and peptidolysis |
| NM_002770 | PRSS2 | 0.0414 | Digestion; proteolysis and peptidolysis |
| D42044 | KIAA0090 | 0.0417 |  |
| NM_007187 | WBP4 | 0.0427 |  |
| NM_025190 | KIAA1641 | 0.0427 |  |
| NM_001894 | CSNK1E | 0.0446 | DNA repair; protein amino acid phosphorylation; signal transduction |
| NM_005630 | SLC21A2 | 0.0459 | Lipid transport |
| AK054901 | MGC2615 | 0.0462 | Transport |
| BC001096 | LOC92689 | 0.0466 |  |
| NM_024565 | FLJ14166 | 0.0470 | Cell cycle; cytokinesis; regulation of cell cycle |
| NM_013378 | VPREB3 | 0.0471 |  |
| NM_001349 | DARS | 0.0474 | Aspartyl-trna aminoacylation; protein biosynthesis; protein complex assembly |
| D63484 | KIAA0150 | 0.0477 |  |
| NM_005612 | REST | 0.0477 | Regulation of transcription, DNA-dependent |
| NM_021158 | C20orf97 | 0.0480 | Apoptosis; protein amino acid phosphorylation |
| NM_004588 | SCN2B | 0.0481 | Ion transport; sodium ion transport; synaptic transmission |
| NM_005554 | KRT6A;KRT6B;KRT6C | 0.0486 | Ectoderm development |
| NM_032827 | FLJ14708 | 0.0493 |  |
| NM_007071 | HHLA3 | 0.0494 |  |
| NM_012385 | P8 | 0.0494 | Cell growth; induction of apoptosis |
| NM_013376 | SEI1 | 0.0497 | Positive regulation of cell proliferation; regulation of cyclin dependent protein kinase activity; regulation of transcription, DNA-dependent |
