# Supplementary material for: Multi-level gene expression profiles affected by thymidylate synthase and 5-fluorouracil in colon cancer
Source: BMC Genomics. 2006 Apr 3;7:68. doi: 10.1186/1471-2164-7-68 (PMC1448211; doi:10.1186/1471-2164-7-68)
Supplement: Additional File 5 — Effect of 5-FU treatment on steady state mRNAs expression in HCT-C18 (TS-) cells. This file contains gene list based on gene expression profiles generated from these samples. These genes are mainly associated with TS independent toxicity in response to 5-FU by direct incorporation to RNA and DNA. The clustering analysis is shown in Figure 6. This file contains a gene list of 185 genes (n = 3, p < 0.05 with 4-fold cut-off) affected by 5-FU exposure in HCT-C18 (TS-) cells in the presence of thymidine. [file 1471-2164-7-68-S5.doc]

# Additional file 5

Effect of 5-FU treatment on steady state mRNAs expression in HCT-C18 (TS-) cells. (185 genes)

| **Genebank access number** | **Gene ID** | **P value** | **Biological function** |
| --- | --- | --- | --- |
| NM_005954 | MT3 | 0.0002 | Cell proliferation; electron transport; metal ion homeostasis; negative regulation of cell growth; negative regulation of dendrite morphogenesis; removal of superoxide radicals; response to hypoxia |
| NM_003695 | E48 | 0.0004 | Cell adhesion |
| NM_023915 | GPR87 | 0.0008 | G-protein coupled receptor protein signaling pathway |
| NM_003125 | SPRR1B | 0.0014 | Epidermis development |
| NM_002658 | PLAU | 0.0019 | Blood coagulation; cell growth and/or maintenance; chemotaxis; negative regulation of blood coagulation; proteolysis and peptidolysis; signal transduction |
| NM_000930 | PLAT | 0.0030 | Blood coagulation; protein modification; proteolysis and peptidolysis |
| NM_005554 | KRT6A;KRT6B;KRT6C | 0.0030 | Ectoderm development |
| NM_017586 | C9orf7 | 0.0034 |  |
| NM_014471 | SPINK4 | 0.0038 |  |
| NM_006762 | LAPTM5 | 0.0041 |  |
| NM_018192 | FLJ10718 | 0.0042 | Protein metabolism |
| NM_021615 | CHST6 | 0.0042 | N-acetylglucosamine metabolism |
| NM_000422 | KRT17 | 0.0044 | Epidermis development |
| NM_004925 | AQP3 | 0.0046 | Excretion; transport |
| AL137708 | SYT8; DKFZp434K0322 | 0.0048 |  |
| NM_000107 | DDB2 | 0.0049 | Nucleotide-excision repair |
| NM_020345 | KBRAS1 | 0.0053 | I-kappab kinase/NF-kappab cascade; small gtpase mediated signal transduction |
| NM_000424 | KRT5 | 0.0055 | Epidermis development |
| NM_005978 | S100A2 | 0.0059 | Biological_process unknown |
| NM_000602 | SERPINE1 | 0.0061 | Blood coagulation |
| NM_000227 | LAMA3 | 0.0061 | Cell adhesion; cell surface receptor linked signal transduction; epidermis development; keratinocyte differentiation |
| NM_005416 | SPRR3 | 0.0063 |  |
| NM_014147 | HSPC047 | 0.0073 |  |
| NM_004881 | PIG3 | 0.0077 | Induction of apoptosis by oxidative stress |
| S73288 | SPRR1A | 0.0078 | Epidermis development |
| NM_000494 | COL17A1 | 0.0079 |  |
| NM_020707 | KIAA1173 | 0.0079 | Biological_process unknown |
| BC033859 | C20orf178 | 0.0080 |  |
| NM_007163 | SLC14A2 | 0.0083 | Urea transport |
| AL137521 | FLJ20255 | 0.0085 | Amino acid transport |
| NM_004924 | ACTN4 | 0.0086 | Cell motility |
| NM_000407 | GP1BB | 0.0094 | Blood coagulation; cell adhesion; cell surface receptor linked signal transduction |
| BC034709 | MGC21116 | 0.0098 | Cell communication; perception of sound |
| NM_000835 | GRIN2C | 0.0099 | Ion transport |
| NM_004073 | CNK | 0.0106 | Cell cycle; protein amino acid phosphorylation; regulation of cell cycle |
| NM_032525 | TUBB-5 | 0.0107 | Microtubule-based movement |
| NM_006142 | SFN | 0.0115 | Cell proliferation; negative regulation of protein kinase activity; regulation of cell cycle; signal transduction |
| NM_025081 | KIAA1305 | 0.0118 |  |
| AL163296 |  | 0.0120 |  |
| NM_015685 | SDCBP2 | 0.0125 | Intracellular signaling cascade; intracellular transport; neurogenesis |
| NM_016580 | PCDH12 | 0.0132 | Cell adhesion; homophilic cell adhesion; neuronal cell recognition |
| NM_001034 | RRM2 | 0.0133 | DNA replication; deoxyribonucleoside diphosphate metabolism |
| NM_000389 | CDKN1A | 0.0137 | Cell cycle arrest; induction of apoptosis by intracellular signals; negative regulation of cell proliferation; regulation of cyclin dependent protein kinase activity |
| NM_019894 | TMPRSS4 | 0.0137 | Proteolysis and peptidolysis |
| NM_000691 | ALDH3A1 | 0.0155 | Aldehyde metabolism; carbohydrate metabolism |
| NM_025106 | FLJ22393 | 0.0158 | Intracellular signaling cascade |
| NM_006989 | CAPRI | 0.0164 | Intracellular signaling cascade |
| NM_000760 | CSF3R | 0.0169 | Cell adhesion; defense response; signal transduction |
| X16354 | CEACAM1 | 0.0170 | Immune response; pregnancy |
| NM_016619 | LOC51316 | 0.0171 |  |
| NM_006932 | SMTN | 0.0177 | Muscle development; smooth muscle contraction |
| AB006627 | ASTN | 0.0184 | Cell adhesion; cell migration; neuronal cell adhesion |
| NM_005597 | NFIC | 0.0187 | DNA replication; regulation of transcription, DNA-dependent; transcription from Pol II promoter |
| NM_020299 | AKR1B10 | 0.0190 | Biological_process unknown |
| NM_021220 | ZNF339 | 0.0191 | Regulation of transcription, DNA-dependent |
| NM_002305 | LGALS1 | 0.0192 | Apoptosis; heterophilic cell adhesion; positive regulation of I-kappab kinase/NF-kappab cascade |
| NM_012320 | LYPLA3 | 0.0193 | Fatty acid metabolism |
| NM_006763 | BTG2 | 0.0194 | DNA repair; negative regulation of cell proliferation |
| NM_020448 | DJ462O23.2 | 0.0194 |  |
| NM_033027 | AXUD1 | 0.0202 | Apoptosis |
| NM_014656 | KIAA0040 | 0.0203 |  |
| NM_002999 | SDC4 | 0.0206 | Biological_process unknown |
| NM_017933 | FLJ20701 | 0.0207 |  |
| X69141 | FDFT1 | 0.0210 | Biosynthesis; cholesterol biosynthesis; isoprenoid biosynthesis; steroid biosynthesis |
| NM_001760 | CCND3 | 0.0216 | Cytokinesis; regulation of cell cycle |
| NM_017490 | EMK1 | 0.0217 | Protein amino acid phosphorylation |
| AB018292 | KIAA0749 | 0.0224 |  |
| NM_015472 | TAZ | 0.0226 | Regulation of transcription, DNA-dependent |
| NM_001236 | CBR3 | 0.0229 | Metabolism |
| NM_006329 | FBLN5 | 0.0230 | Blood coagulation; cell-matrix adhesion |
| NM_006088 | TUBB2 | 0.0237 | Microtubule polymerization; microtubule-based movement |
| NM_018986 | FLJ20356 | 0.0237 |  |
| NM_014421 | DKK2 | 0.0239 | Wnt receptor signaling pathway; development; negative regulation of Wnt receptor signaling pathway |
| BM465550 | MAP4 | 0.0239 |  |
| AI820701 |  | 0.0248 |  |
| NM_003236 | TGFA | 0.0248 | Cell proliferation; cell-cell signaling; regulation of cell cycle |
| NM_003504 | CDC45L | 0.0254 | DNA replication; DNA replication checkpoint; DNA replication initiation; regulation of cell cycle |
| NM_033256 | PPP1R14A | 0.0257 |  |
| BQ670872 | FAM20C | 0.0259 |  |
| NM_032461 | SPANXB1;SPANXC | 0.0261 | Spermatid cell development |
| AL137513 | LOC150568 | 0.0264 |  |
| NM_006302 | GCS1 | 0.0268 | N-linked glycosylation; carbohydrate metabolism; oligosaccharide metabolism |
| NM_003040 | SLC4A2 | 0.0271 | Anion transport |
| NM_007076 | HYPE | 0.0272 | Regulation of cell cycle |
| NM_006083 | IK | 0.0275 | Cell-cell signaling; immune response |
| NM_016433 | GLTP | 0.0277 | Lipid transport |
| NM_012144 | DNAI1 | 0.0277 |  |
| NM_001200 | BMP2 | 0.0279 | Cell growth and/or maintenance; cell-cell signaling; skeletal development |
| M22637 | LYL1 | 0.0280 |  |
| NM_004166 | SCYA14;SCYA15 | 0.0284 | Calcium ion homeostasis; immune response; positive regulation of cell proliferation |
| AB028949 | KIAA1026 | 0.0289 |  |
| NM_023938 | MGC2742 | 0.0290 |  |
| NM_001069 | TUBB | 0.0297 |  |
| NM_003258 | TK1 | 0.0297 | DNA metabolism |
| NM_022772 | EPS8R2 | 0.0299 |  |
| NM_012189 | FSP-2 | 0.0300 | Signal transduction |
| AK054606 | ZNF79 | 0.0302 | Regulation of transcription, DNA-dependent |
| AA147817 |  | 0.0303 |  |
| NM_001419 | ELAVL1 | 0.0304 | RNA catabolism; development |
| NM_006613 | GRAP | 0.0311 | RAS protein signal transduction; cell-cell signaling; intracellular signaling cascade |
| NM_002355 | M6PR | 0.0318 | Endosome to lysosome transport; receptor mediated endocytosis |
| NM_031477 | MGC10500 | 0.0319 |  |
| BE049190 | MAZ | 0.0322 |  |
| AL133645 | LOC90133 | 0.0324 |  |
| NM_032377 | MGC4549 | 0.0324 |  |
| NM_003282 | TNNI2 | 0.0327 | Muscle development; regulation of muscle contraction |
| NM_020770 | CGN | 0.0327 | Biological_process unknown |
| AF068624 | ALAS2 | 0.0330 |  |
| NM_019099 | LOC55924 | 0.0337 |  |
| U85658 | TFAP2C | 0.0346 | Cell-cell signaling; regulation of transcription from Pol II promoter |
| BE799837 |  | 0.0352 |  |
| AK055031 | FLJ30469 | 0.0353 |  |
| U61084 | APOBEC3B; ARP4; ARCD3; PHRBNL; APOBEC1L; FLJ21201; DJ742C19.2 | 0.0360 |  |
| NM_001333 | CTSL2 | 0.0361 | Proteolysis and peptidolysis |
| NM_002275 | KRT15 | 0.0363 | Epidermis development |
| NM_004419 | DUSP5 | 0.0364 | Protein amino acid dephosphorylation |
| NM_006518 | SPRR2C | 0.0364 |  |
| NM_003844 | TNFRSF10A | 0.0365 | Apoptosis; caspase activation; induction of apoptosis; induction of apoptosis via death domain receptors; signal transduction |
| NM_007371 | BRD3 | 0.0365 | Biological_process unknown |
| NM_018494 | LRDD | 0.0371 | Signal transduction |
| AF229181 | SBA2 | 0.0375 | Intracellular signaling cascade |
| NM_152718 | FLJ32009 | 0.0376 |  |
| NM_005953 | MT2A; | 0.0379 | Copper ion homeostasis |
| NM_032510 | PARD6G | 0.0382 | Cell cycle; cytokinesis |
| NM_024302 | MMP28 | 0.0386 | Proteolysis and peptidolysis |
| NM_005009 | NME4 | 0.0389 | CTP biosynthesis; GTP biosynthesis; UTP biosynthesis; nucleoside metabolism |
| NM_018992 | FLJ20040 | 0.0389 | Potassium ion transport |
| NM_033212 | MGC10992 | 0.0391 |  |
| NM_004704 | U3-55K | 0.0403 | Rrna processing |
| AF223391 |  | 0.0405 |  |
| NM_033285 | TP53INP1 | 0.0406 |  |
| NM_020529 | NFKBIA | 0.0417 | Apoptosis; cytoplasmic sequestering of NF-kappab; response to pathogenic bacteria |
| NM_001182 | ALDH7A1 | 0.0421 | Aldehyde metabolism; perception of sound |
| NM_014668 | KIAA0575 | 0.0429 | Biological_process unknown |
| NM_002653 | PITX1 | 0.0430 | Morphogenesis; regulation of transcription, DNA-dependent; skeletal development |
| NM_007274 | HBACH | 0.0430 | Lipid metabolism |
| BC002829 | S100A2 | 0.0432 | Biological_process unknown |
| NM_016381 | TREX1 | 0.0434 | DNA recombination; DNA replication; mismatch repair |
| AB033093 | DKFZP727C091 | 0.0438 |  |
| NM_001665 | ARHG | 0.0438 | Rho protein signal transduction; positive regulation of cell proliferation; regulation of cell cycle |
| NM_018960 | GNMT | 0.0440 | Protein modification |
| NM_002176 | IFNB1 | 0.0440 | B-cell proliferation; anti-inflammatory response; caspase activation; cell surface receptor linked signal transduction; defense response; natural killer cell activation; negative regulation of cell proliferation; negative regulation of virion penetration; positive regulation of innate immune response; regulation of MHC class I biosynthesis; response to virus |
| NG_001336 | TRG | 0.0443 |  |
| NM_004583 | RAB5C | 0.0443 | Intracellular protein transport; small gtpase mediated signal transduction |
| NM_032270 | DKFZp586J1119 | 0.0445 |  |
| NM_001988 | EVPL | 0.0447 | Epidermis development |
| NM_002855 | PVRL1 | 0.0447 | Cell-cell adhesion; immune response; viral entry |
| BC011762 | CYFIP2 | 0.0448 | Immune response |
| NM_003334 | UBE1 | 0.0450 | DNA replication; ubiquitin cycle |
| NM_014877 | HELZ | 0.0450 |  |
| NM_004295 | TRAF4 | 0.0451 | Apoptosis; development |
| X03069 | HLA-DRB1 | 0.0452 |  |
| NM_015456 | COBRA1 | 0.0452 | Regulation of transcription, DNA-dependent |
| NM_052815 | IER3 | 0.0453 | Anti-apoptosis; cell growth and/or maintenance; morphogenesis |
| NM_000693 | ALDH1A3 | 0.0454 | Alcohol metabolism; lipid metabolism |
| NM_002450 | MT1L | 0.0455 |  |
| BE547641 | RAVER1 | 0.0456 |  |
| NM_002949 | MRPL12 | 0.0457 | Protein biosynthesis |
| NM_032583 | ABCC11 | 0.0457 | Transport |
| NM_022776 | OSBPL11 | 0.0458 | Lipid transport; steroid metabolism |
| NM_005853 | IRX5 | 0.0459 | Regulation of transcription, DNA-dependent |
| NM_032457 | PCDH7 | 0.0459 | Cell adhesion; homophilic cell adhesion |
| NM_033133 | CNP | 0.0459 | Cyclic nucleotide catabolism; synaptic transmission |
| NM_003273 | TM7SF2 | 0.0468 | Cholesterol biosynthesis |
| NM_016229 | LOC51700 | 0.0470 | Electron transport |
| NM_019109 | HMT-1 | 0.0472 | Biosynthesis |
| NM_002640 | SERPINB8 | 0.0474 |  |
| NM_016551 | TM7SF3 | 0.0474 |  |
| NM_024417 | FDXR | 0.0474 | Cholesterol metabolism; electron transport; energy pathways; steroid biosynthesis |
| NM_006502 | POLH | 0.0476 | Regulation of DNA repair |
| NM_013376 | SEI1 | 0.0477 | Positive regulation of cell proliferation; regulation of cyclin dependent protein kinase activity; regulation of transcription, DNA-dependent |
| NM_025069 | FLJ14299 | 0.0480 |  |
| NM_004870 | MPDU1 | 0.0481 |  |
| NM_014029 | HSPC022 | 0.0481 |  |
| NM_003240 | EBAF | 0.0482 | Cell growth; cell-cell signaling; oocyte axis determination; transforming growth factor beta receptor signaling pathway |
| NM_006162 | NFATC1 | 0.0486 | Regulation of transcription, DNA-dependent; transcription from Pol II promoter |
| NM_004628 | XPC | 0.0486 | Nucleotide-excision repair |
| NM_014999 | RAB21 | 0.0487 | Intracellular protein transport; small gtpase mediated signal transduction |
| NM_001453 | FOXC1 | 0.0487 | Morphogenesis; regulation of transcription, DNA-dependent; visual perception |
| NM_005326 | HAGH | 0.0489 |  |
| NM_006516 | SLC2A1 | 0.0489 | Carbohydrate transport; glucose transport |
| NM_002638 | PI3 | 0.0490 | Copulation |
| NM_002722 | PPY | 0.0491 | Digestion; protein secretion |
| AJ303098 | PI4KII | 0.0499 | Phosphatidylinositol biosynthesis |
| NM_005643 | TAF11 | 0.0500 | Regulation of transcription; regulation of transcription, DNA-dependent |
